# Direct replication of Gervais & Norenzayan (2012): No evidence that analytic thinking decreases religious belief

**DOI:** 10.1371/journal.pone.0172636

**Published:** 2017-02-24

**Authors:** Clinton Sanchez, Brian Sundermeier, Kenneth Gray, Robert J. Calin-Jageman

**Affiliations:** 1 Department of Psychology, Dominican University, River Forest, Illinois, United States of America; 2 School of Education, DePaul University, Chicago, Illinois, United States of America; 3 Department of Psychology, Concordia University Chicago, River Forest, Illinois, United States of America; 4 Department of Psychology, College of DuPage, Glen Ellyn, Illinois, United States of America; Universiteit van Amsterdam, NETHERLANDS

## Abstract

Gervais & Norenzayan (2012) reported in *Science* a series of 4 experiments in which manipulations intended to foster analytic thinking decreased religious belief. We conducted a precise, large, multi-site pre-registered replication of one of these experiments. We observed little to no effect of the experimental manipulation on religious belief (*d* = 0.07 in the wrong direction, 95% CI[-0.12, 0.25], *N* = 941). The original finding does not seem to provide reliable or valid evidence that analytic thinking causes a decrease in religious belief.

## Introduction

Religion seems to be a cultural universal and yet there are marked individual differences in degrees of religious belief and practice. One factor that might explain individual variation in religious faith could be a proclivity for intuitive styles of cognition over more analytic/reflective modes of cognition. This possibility was recently explored in Gervais & Norenzayan (G&N) in a paper published in *Science* [1]. Specifically, G&N reported a weak negative correlation between the tendency to engage in analytic thinking and belief in God. Moreover, G&N reported four experiments in which manipulations meant to increase analytic thinking substantially reduced self-reported religious belief.

Since publication, concerns have emerged about these findings. Specifically, an analysis of psychology papers published in *Science* flagged the paper by G&N [2] for failing a test for excess significance [3]. However, there is spirited debate about whether or not tests of excess significance can be meaningfully interpreted at the level of individual papers [4].

To provide an unbiased estimate of the degree to which manipulating analytic thinking affects religious belief we conducted a precise, large, pre-registered replication of Study 2 of G&N. In this study, participants were randomly assigned to view images of Rodin’s *The Thinker* (*n* = 26) or Myron’s *Discobolus* (*n* = 31). The images of *The Thinker* were intended to prime analytic thinking; the images of *Discobolus* were used as a neutral control. Immediately after viewing the images, all participants were asked to report their belief in God on a scale from 0 to 100. In the original study, participants exposed to *The Thinker* reported lower belief in God as compared to participants exposed to *Discobolus* (*d* = -0.59).

To ensure our results did not reflect idiosyncrasies of a particular participant pool, we collected participants from three different schools varying in religious affiliation (a public community college, a private Catholic university, and a private Lutheran university) as well as from an online Internet marketplace (U.S.-based workers from Mechanical Turk).

Before conducting this study we completed a replication recipe [5], in which we specified our sampling plan, hypotheses, analysis plan, and materials. We also implemented quality controls to ensure suitable participants, and included an additional positive control study to check the quality of the replication [6,7]. Furthermore, we asked the first author of the original study to review our materials (Will Gervais, personal communication), and with his gracious cooperation ensured that our materials were precisely matched to the original. We then pre-registered our plan on the Open Science Framework prior to any data collection. All our materials, raw data, and analysis files can be found there (https://osf.io/qc6rh/).

This manuscript is a complete report of all the studies we have done attempting to replicate the effects of analytic thinking on religious belief. We report how we determined our sample size, all data exclusions (if any), all manipulations, and all measures in the study [8].

## Methods

### Sampling plan

We set a sample-size target of 227 participants per condition. This was based on the largest sample size used by G&N (Study 5, *n* = 91 in the analytic group) and the recommendation of achieving at least 2.5 times the original sample size [9].

### Participants

Participants at the school sites were recruited from Psych 101 participant pools, and received course credit for completing the study. For the online sample, participants were recruited via Amazon’s Mechanical Turk service (https://requester.mturk.com) and paid $0.50 USD for completing the study. Recruitment was restricted to U.S. based participants with a lifetime HIT approval rate > 90%. For all participants, informed consent was obtained by button press; this procedure was approved by the Dominican University IRB Committee (Application 2014–69) and at each of the other sites where student data was collected.

At the community college site, 220 participants completed the study. Of these, one participant guessed the hypothesis, leaving 219 for analysis. At the private Lutheran university, 157 participants completed the study. Of these, 1 guessed the hypothesis, leaving 156 for analysis. At the private Catholic university, 166 participants completed the study. None of these guessed the hypothesis. In the online study, 491 completed the study. Of these, 80 failed one or more of our pre-registered quality controls (see below), leaving 411 for analysis. Table 1 presents demographic information for each sample.

**Table 1:** Participant demographics.

	Age	% Male	% Black or African-American	% East Asian	% Hispanic or Latino/a	% South Asian	% White or Caucasian	% Other
Original Study	20.4	32.0	0.0	50.0	0.0	12.0	30.0	8.0
All Replication Sites	26.7	42.4	6.3	3.0	17.4	3.0	61.9	4.8
U.S. Online	35.4	51.0	7.1	3.6	4.1	1.7	81.5	1.9
Community college	20.0	38.0	2.7	4.6	14.2	7.8	61.2	9.1
Private Catholic	20.0	39.8	4.8	1.2	45.8	0.0	37.3	11.4
Private Lutheran	20.1	38.5	11.5	1.9	29.5	0.0	51.3	5.7

*Note*: Other collapses those who marked “Other” as well as those who marked “American Indian or Alaska Native” or “Native Hawaiian or Pacific Islander” (under 2% in each sample). Some participants gave no response.

#### Materials and procedure

We used the exact same measures as in Study 2 of G&N, matching not only the images and dependent measures, but also the cover story, instructions, and presentation sequence. As in the original study, the entire experiment was run via a computer interface. Participants at the school sites completed their studies in a lab setting.

#### Instructional manipulation check

Online participants were first directed to a pre-screening survey. This consisted of a single-item instructional manipulation check [10]. See the materials posted online for details.

#### Cover story

Onscreen instructions informed participants that they would participate in three separate “mini-studies”. At the outset of the study, each participant was presented with a multiple choice item with three options and told to select one to randomly determine which of the three mini-studies would be presented first. Regardless of which option was selected, the manipulation of analytic thinking was presented next. However, to maintain the cover story, this section was always labelled “Study X” where X was the option number of the mini-study the participant had selected. After the manipulation, participants selected one of the two remaining mini-studies. Regardless of their choice, the main dependent measure (belief in God) was presented next followed by questions for age, gender, and ethnicity. Finally, participants were told they would complete the last mini-study, which consisted of the positive control and remaining demographic and quality control items.

#### Manipulation of analytic thinking

Participants were told they were participating in a mini-study consisting of a memory task and that they would view four images for 30s each. They were then randomly assigned to view either four images of *The Thinker* or four images of *Discobolus* (same images as in the original study). Each image was presented for 30s.

#### Measure of religious belief

Participants were told that the next mini-study collected basic demographic information. The next page then gave this open-ended prompt:

How strongly do you believe in God (from 0–100)? To clarify, if you are certain that God does not exist, please put "0" and if you are certain that God does exist, then put "100."

The response box was set to reject non-numeric and out-of-range responses.

#### Demographic information

Following the measure of religious belief, all participants reported their age, gender, religion, and ethnicity (one question per page) using the same prompts as in the original study.

#### Positive control

To help indicate the overall quality of our replication we included as a positive control an additional experiment with a well-defined effect size. Specifically, we used the retrospective gambler’s task [11]. In this task, participants were asked to imagine entering a casino where they observe a gambler roll three dice and obtain either a) two sixes (the *all-sixes* condition) or b) two sixes and a three (the *some-sixes* condition). After imagining the scenario, participants were asked to estimate how many times the gambler had already rolled the dice (open-ended response). The expected effect is for those who read the *all-sixes* scenario to estimate more prior rolls than those who read the *some-sixes* scenario. We obtained the materials for this task from the Many Labs project [12].

Group assignment to the positive control was made randomly and independent of image condition. As in the Many Labs project, we dealt with the non-normal distribution of estimated rolls by applying a square-root transformation prior to analysis.

#### Memory manipulation check

We added a memory manipulation check to measure the degree to which participants attended to the priming images. Participants were presented with the entire set of eight images (four *Thinker*, four *Discobolus*) and asked to put a mark next to all and only the images they had seen in the initial mini study. The order of images was randomized for each participant.

#### Additional measures for quality controls

We also added items asking participants to report a) if they spoke English as their first and primary language or not, b) if they were currently living in the U.S.A. or not. For the online participants these measures were made with an assurance that payment would be received regardless of how they were answered.

#### Suspicion check

As in the original study, all participants were given an open ended prompt “If you had to guess, what do you think was the hypothesis for this study?” Responses were reviewed by two of the authors independently. Any response which specifically mentioned both religion and thinking/cognition/reasoning was classified as a correct guess.

### Exclusions

In the original study, only participants who guessed the research hypothesis were excluded from analysis. We adopted the same criteria for our school samples, and this led to the exclusion of 2 participants.

For our online study, we pre-registered a number of additional exclusion criteria to ensure that participants would be a) similar in location and language to those in the original study and b) attentive to the study. Specifically, we removed:

- 11 participants who used an IP address that resolved to an address outside the U.S.A.
- An additional 5 participants who failed the instructional manipulation check more than two times.
- An additional 11 participants who classified themselves as non-native English speakers
- No additional participants for self-reporting being currently outside of the U.S.A.
- An additional 16 participants who took an unusually short or long time to complete the study. Specifically, we calculated the median amount of time for study completion and then excluded participants who took less than 40% (unusually quick responses) or more than 250% (unusually slow responses) of the median study duration.
- An additional 4 participants who gave an outlier response (|z| > 4) on the positive control, the retrospective gambler’s task. A similar exclusion criteria was used in the Many Labs study of this task [13] to ensure exclusion of non-serious responding.
- An additional 32 participants who failed the memory manipulation check (more than 1 incorrect choice)
- An additional participant who guessed the hypothesis in the suspicion check.

Excluded participants are not represented in any of the results presented here, but essentially identical results are obtained without these exclusions.

## Results

We found no effect of priming analytic thinking on religious belief (*F*(1,944) = 0.78, *p* = .38, *η*^2^ = 0.001) nor any interaction between collection site and type of prime (*F*(3,944) = 1.22, *p* = .30, *η*^2^ = 0.004). Analyzing each sample separately (Table 2), the effect was not significant in the online sample (*t*(409) = -0.59, *p* = 0.56), at the community college (*t*(217) = - 0.17, *p* = 0.86), nor at the private Catholic university (*t*(164) = .38, *p* = 0.71). The effect at the private Lutheran university reached statistical significance, but in the opposite direction expected (*t*(154) = 2.41, *p* = 0.02). When synthesizing results across all 4 study locations using a random-effects meta-analysis, our data is consistent with little to no effect of the experimental manipulation on religious belief (Table 1, bottom row).

**Table 2:** Effect of visual priming for analytic thinking on religious belief.

	N_D_/N_T_	*Discobolus M* (*SD*)	*Thinker M* (*SD*)	Cohen’s *d* with 95% CI
Original Study	31/26	61.6 (35.7)	41.4 (31.5)	-0.59 [-1.12, -0.06]
Direct Replication				
U.S. Online	199/212	50.6 (43.5)	48.1 (42.3)	-0.06 [-0.25, 0.14]
Community college	110/109	66.7 (36.8)	65.9 (36.9)	-0.02 [-0.29, 0.24]
Private Catholic	81/85	76.6 (32.6)	78.8 (31.2)	0.06 [-0.24, 0.36]
Private Lutheran	83/73	76.9 (29.5)	87.4 (26.1)	0.38 [0.07, 0.70]
**All Replication Sites**	473/479			**0.07 [-0.12, 0.25]**

*Note*: *N*_D_/*N*_T_ reports sample size for *Discobolus* and *Thinker* conditions, respectively. Religious belief was measured on a scale from 0 to 100. The bottom row reports the integrated effect size over all the replication sites using a random-effects meta-analysis [14]. A test for heterogeneity of effect sizes was not significant (Q(3) = 5.73, *p* = 0.13).

Our inability to detect a substantial decline in religious belief was not due to quality issues as we detected an expected effect in an additional positive control study with the same participants (*F*(1,963) = 21.2, *p* < .001, Table 3).

**Table 3:** Retrospective Gambler’s fallacy.

	N/N	Some Sixes *M* (*SD)*	All Sixes *M* (*SD*)	Cohen’s *d*
U.S. Online sample	411	2.3 (1.9)	3.2 (3.0)	0.34 [0.14, 0.53]
Community college	228	1.6 (1.5)	2.5 (2.1)	0.49 [0.23, 0.76]
Private Catholic	173	1.7 (1.9)	2.3 (2.6)	0.30 [0.00, 0.60]
Private Lutheran	159	2.1 (2.1)	2.7 (2.4)	0.26 [-0.06, 0.57]
**All Replication Sites**				**0.35 [0.23, 0.48]**

*Note*: In the Retrospective Gambler’s fallacy participants are asked to imagine entering a casino and witnessing a gambler roll three dice, obtaining either three sixes (*all sixes* condition) or two sixes and a three (*some sixes* condition). They are then asked to estimate how many rolls the gambler had previously made. The classic effect is for participants to estimate more previous rolls in the all sixes condition than in the some sixes condition. Data reported here are estimated number of rolls after square-root transformation, which is that standard method of analysis for this effect [6,11,13]. The bottom row reports the integrated effect size over all the replication sites using a random-effects meta-analysis [14]. A test for heterogeneity of effect size was not significant: *Q*(3) = 1.59, *p* = 0.66.

We found the measure of religious belief to be strongly bi-modal (mostly believers and non-believers). We thus explored alternative methods of analysis for the dependent measure (e.g., Chi Square) but found essentially identical results (e.g., χ^2^(1) = 0.25, *p* = 0.62, *d* = 0.03 for the association between condition and a median split of religious belief).

We did note that the largest effect we observed (in the wrong direction) was at the site with the highest level of religious belief in the control group. This could represent normal sampling variability. We wondered, though, if religious belief might function as a moderator. Specifically, it could be that analytic thinking simply reinforces existing tendencies, driving those with moderate religious belief to less strong belief, but those with strong belief to even stronger belief. To examine this possibility we conducted a meta-regression over our study sites and the original study [14]. As a predictor of effect size, we used the average belief in each control group, reasoning that this is a reasonable proxy for the typical strength of belief in each sample. We found that average belief in each control group is not a statistically significant predictor of effect size (β = .38, 95% CI [-0.04, 0.80], *p* = 0.08, *N* = 5 study sites; see S1 Fig). More convincingly, G&N directly tested for possible moderation by religious belief by conducting studies in which belief was measured innocuously well before the experimental protocol. In these more sensitive tests conducted at the participant level they found no evidence for moderation by religious belief (regression weights for belief x condition interaction: β = -.04, *p* = 0.64, *N* = 93 for Study 3; β = .02, *p* = 0.65, *N* = 153 for Study 5). Thus, moderation by religious belief does not seem like a promising explanation for the results we obtained.

## Discussion

Study 2 of G&N found that exposure to *The Thinker* produced a moderate-to-large decline in religious belief (*d* = -0.59). In contrast, our replication of this study found little to no effect of the experimental manipulation (*d* = 0.07, 95% CI[-0.12, 0.25]). The overall confidence interval we obtained is centered close to 0 and consistent with no more than a very small effect in the expected direction.

What might explain the notable difference between our results and those reported by G&N? We can rule out substantive differences in materials and procedures, as these were essentially identical. We can also rule out idiosyncrasies in participant pools, as we collected diverse samples and used extensive quality controls. Finally, we can also rule out researcher incompetence, as we were able to detect an expected effect of similar size using a positive control.

One possibility is that Study 2 of G&N substantially over-estimated the effect of the manipulation on religious belief. This seems likely, not only because of the data presented here but also because evidence published while this project was in progress suggests that the experimental manipulation may not actually influence analytic thinking. Although G&N reported in supplemental materials that exposure to *The Thinker* substantially increases scores on a syllogistic reasoning task (*d* = 0.86, 95% CI[0.23, 1.53], *N* = 40), a much larger study [15] estimates that this manipulation produces little to no change in scores on the Cognitive Reflection Test (CRT), another measure of analytic thinking (Study 5: *d* = -0.04, 95% CI[-0.29, .21], N = 247). In fact, concerns have now emerged about the construct validity of all the manipulations used in the experiments reported by G&N. Studies 3 and 4 used a verbal fluency task priming procedure [2]. Although a pilot study reported in supplemental materials indicated that this procedure substantially improves analytic thinking on the “Moses Illusion” (*d* = 1.04, 95% CI[0.49, 1.6], *N* = 40), two large studies [15] now estimate little to no effect on the CRT (Studyes 2 and 3: *d* = 0.03, 95% CI [-0.11, 0.16], N = 846. Finally, Study 5 of G&N used verbal disfluency to prime analytic thinking, but a large-scale study [16] shows this manipulation produces no meaningful change on the CRT (*d* = -0.01, 95% CI[-0.06, 0.04], *N* = 7,367).

Based on our results and the notable issues of construct validity that have emerged we conclude that the experiments reported by G&N do not provide strong evidence that analytic thinking causes a reduction in religious belief. This conclusion is further supported by results from an independent set of conceptual replications that was recently published [17] which also found little to no effect of analytic thinking manipulations on religious belief. On the other hand, a successful direct replication of Study 4 of G&N has been reported with Turkish undergraduates [18] and a conceptually similar experiment has also found that analytic thinking decreases religious belief [19].

Although the experimental literature is mixed, the weak negative correlation between analytic thinking and religious belief reported by G&N has now been supported by additional replications [20,21] and a meta-analyses [22]. This correlational data is not only suggestive; it is also informative for planning experiments to test causal links between these constructs. Specifically, meta-analysis suggests the relationship between analytic thinking and religious belief is reliable but weak: *r* = -0.18 [22]. Generously assuming that the observed correlation exclusively reflects a causal relationship from analytic thinking to decreased religious belief, this indicates that each 1 standard deviation increase in analytic thinking should be expected to produce, on average, only a 0.18 standard deviation decline in religious belief. Thus, experiments intended to test this causal pathway must either a) utilize extremely impactful manipulations of analytic thinking or b) obtain sample sizes large enough to reliably detect very subtle shifts in religious belief. Another promising strategy might be to conduct longitudinal research examining the temporal relationships between changes in these two constructs. For example, the robust literature on the effects of college education on religious belief both in the U.S. [23] and internationally [24] provide good models for tracking the inter-play between analytic thinking and religious belief over time.

## Supporting information

S1 Fig Exploration of control belief as a moderator of the effect of analytic thinking on religious belief. This scatterplot shows a meta-regression encompassing our 4 replication sites (4 large circles) and the original study (small circle). On the Y axis is the standardized effect size observed (negative values indicate decreased religious belief in the group primed for analytic thinking). On the X axis is the average level of religious belief reported in the control group. Control belief level was not a statistically significant predictor of effect size (unstandardized slope = 0.10, 95% CI[-0.001, 0.021], *p* = 0.08). Although the CI is very broad, additional evidence from G&N strongly suggests that religious belief does not strongly moderate the effect of analytic thinking.
(PNG)

## References

[1] Gervais WM, Norenzayan A. Analytic Thinking Promotes Religious Disbelief. Science (80-). 2012;336: 493–496. 10.1126/science.1215647 22539725

[2] Gervais WM, Norenzayan A. Analytic Thinking Promotes Religious Disbelief. Science (80-). 2012;336: 493–496. 10.1126/science.1215647 22539725

[3] Francis G, Tanzman J, Matthews WJ. Excess success for psychology articles in the journal science. PLoS One. 2014;9: e114255 10.1371/journal.pone.0114255 25474317 PMC4256411

[4] Simonsohn U. It Does Not Follow: Evaluating the One-Off Publication Bias Critiques by Francis (2012a, 2012b, 2012c, 2012d, 2012e, in press). Perspect Psychol Sci. 2012;7: 597–599. 10.1177/1745691612463399 26168117

[5] Brandt MJ, IJzerman H, Dijksterhuis A, Farach FJ, Geller J, Giner-Sorolla R, et al The replication recipe: What makes for a convincing replication? J Exp Soc Psychol. The Authors; 2014;50: 217–224.

[6] Cusack M, Vezenkova N, Gottschalk C, Calin-Jageman RJ. Direct and conceptual replications of Burgmer & Englich (2012): Power may have little to no effect on motor performance. Haddad JM, editor. PLoS One. 2015;10: e0140806 10.1371/journal.pone.0140806 26536592 PMC4633206

[7] Moery E, Calin-Jageman RJ. Direct and conceptual replications of eskine (2013): Organic food exposure has little to no effect on moral judgments and prosocial behavior. Soc Psychol Personal Sci. 2016;7: 312–319.

[8] Simmons JP, Nelson LD, Simonsohn U. A 21 word solution. SSRN Electron J. 2012; 1–4.

[9] Simonsohn U. Small telescopes: detectability and the evaluation of replication results. Psychol Sci. 2015;26: 559–569. 10.1177/0956797614567341 25800521

[10] Oppenheimer DM, Meyvis T, Davidenko N. Instructional manipulation checks: Detecting satisficing to increase statistical power. J Exp Soc Psychol. Elsevier Inc.; 2009;45: 867–872.

[11] Oppenheimer DM, Monin B. The retrospective gambler’s fallacy: Unlikely events, constructing the past, and multiple universes. Judgm Decis Mak. 2009;4: 326–334.

[12] Klein RA, Ratliff KA, Vianello M, Adams RB, Bahník Š, Bernstein MJ, et al Investigating variation in replicability: A “Many Labs” replication project. Soc Psychol (Gott). 2014;45: 142–152.

[13] Klein RA, Ratliff KA, Vianello M, Adams RB, Bahník Š, Bernstein MJ, et al Investigating variation in replicability. Soc Psychol (Gott). 2014;45: 142–152.

[14] Wallace BC, Dahabreh IJ, Trikalinos T a, Lau J, Trow P, Schmid CH. Closing the gap between methodologists and end-Users: R as a computational back-end. Wiley Interdiscip Rev Comput. 2012;49: 1–15.

[15] Deppe KD, Gonzalez FJ, Neiman JL, Jacobs C, Pahlke J, Smith KB. Reflective liberals and intuitive conservatives: A look at the Cognitive Reflection Test and ideology. Judgm Decis Mak. 2015;10: 314–331.

[16] Meyer A, Frederick S, Burnham TC, Guevara Pinto JD, Boyer TW, Ball LJ, et al Disfluent fonts don’t help people solve math problems. J Exp Psychol Gen. 2015;144: e16–e30. 10.1037/xge0000049 25844628

[17] Yonker JE, Edman LRO, Cresswell J, Barrett JL. Primed Analytic Thought and Religiosity: The Importance of Individual Characteristics. 2016;8: 298–308.

[18] Yilmaz O, Karadöller DZ, Sofuoglu G. Analytic Thinking, Religion and Prejudice: An Experimental Test of the Dual-Process Model of Mind. Int J Psychol Relig. Routledge; 2016;8619: 10508619.2016.1151117.

[19] Shenhav A, Rand DG, Greene JD. Divine intuition: Cognitive style influences belief in God. J Exp Psychol Gen. 2012;141: 423–428. 10.1037/a0025391 21928924

[20] Pennycook G. Evidence that analytic cognitive style influences religious belief: Comment on Razmyar and Reeve (2013). Intelligence. Elsevier Inc.; 2014;43: 21–26.

[21] Gervais WM. Override the controversy: Analytic thinking predicts endorsement of evolution. Cognition. Elsevier B.V.; 2015;142: 312–321. 10.1016/j.cognition.2015.05.011 26072277

[22] Pennycook G, Ross RM, Koehler DJ, Fugelsang JA. Atheists and agnostics are more reflective than religious believers: Four empirical studies and a meta-analysis. PLoS One. 2016;11: 1–18. 10.1371/journal.pone.0153039 PMC4824409 27054566

[23] Mayrl D, Uecker JE. Higher Education and Religious Liberalization among Young Adults. Soc Forces. 2011;90: 181–208. 10.1093/sf/90.1.181 22665942 PMC3365855

[24] Schwadel P. Explaining Cross-National Variation in the Effect of Higher Education on Religiosity. J Sci Study Relig. 2015;54: 402–418.

