# Supplementary figures and images for: Direct replication of Gervais & Norenzayan (2012): No evidence that analytic thinking decreases religious belief
Source: PLoS One. 2017 Feb 24;12(2):e0172636. doi: 10.1371/journal.pone.0172636 (PMC5325262; doi:10.1371/journal.pone.0172636)

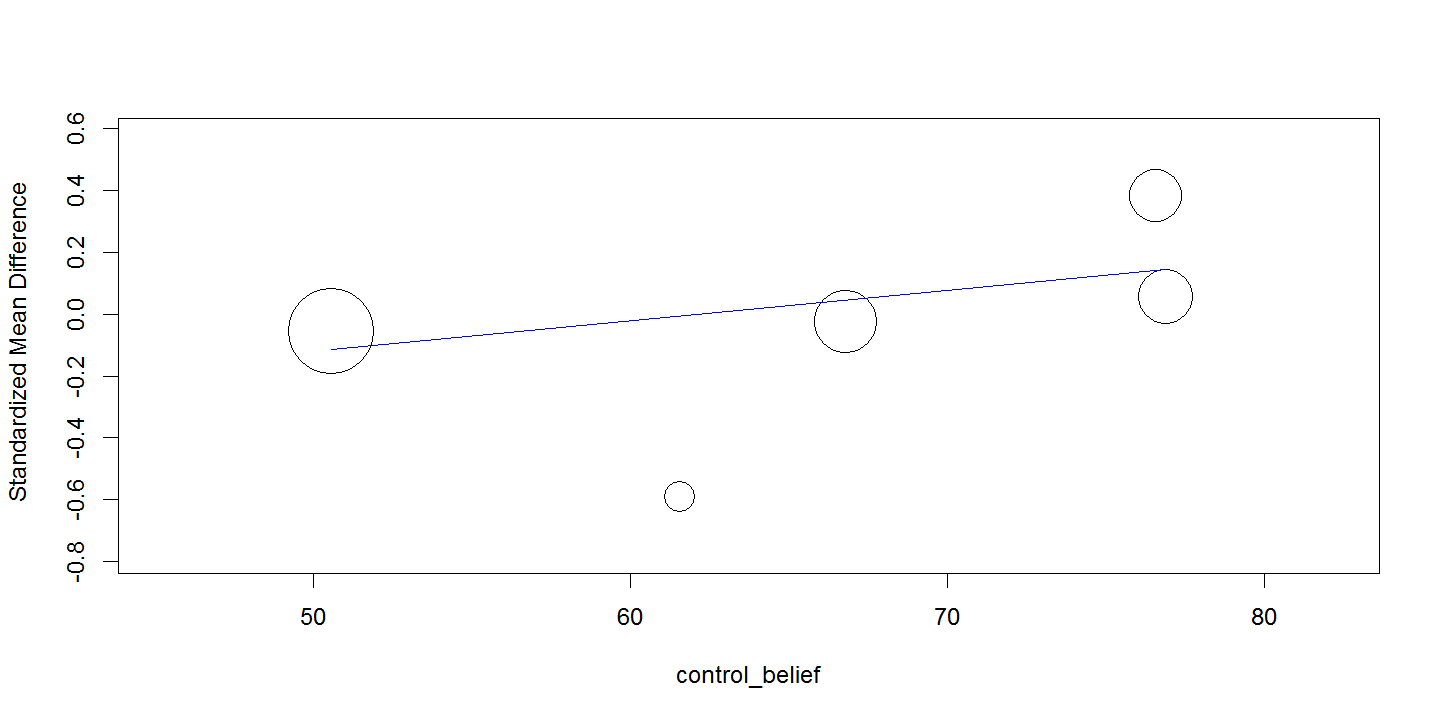

Supplement: S1 Fig — This scatterplot shows a meta-regression encompassing our 4 replication sites (4 large circles) and the original study (small circle). On the Y axis is the standardized effect size observed (negative values indicate decreased religious belief in the group primed for analytic thinking). On the X axis is the average level of religious belief reported in the control group. Control belief level was not a statistically significant predictor of effect size (unstandardized slope = 0.10, 95% CI[-0.001, 0.021], p = 0.08). Although the CI is very broad, additional evidence from G&N strongly suggests that religious belief does not strongly moderate the effect of analytic thinking. (PNG) [file pone.0172636.s001.png]
